# Interstellar photovoltaics

**DOI:** 10.1038/s41598-023-43224-5

**Published:** 2023-09-26

**Authors:** Nora Schopp, Ernazar Abdikamalov, Andrii I. Mostovyi, Hryhorii P. Parkhomenko, Mykhailo M. Solovan, Ernest A. Asare, Guillermo C. Bazan, Thuc-Quyen Nguyen, George F. Smoot, Viktor V. Brus

**Affiliations:** 1 Center for Polymers and Organic Solids, Department of Chemistry and Biochemistry, University of California Santa Barbara (UCSB), Santa Barbara, CA 93106 USA; 2 Department of Physics, School of Sciences and Humanities, Nazarbayev University, 010000 Astana, Republic of Kazakhstan; 3 Energetic Cosmos Laboratory, Nazarbayev University, Astana, 010000 Republic of Kazakhstan; 4 Department of Electronics and Energy Engineering, Yuriy Fedkovych Chernivtsi National University, Chernivtsi, 58012 Ukraine; 5 Faculty of Physics, Adam Mickiewicz University, 61-614 Poznan, Poland; 6 Departments of Chemistry and Chemical & Biomolecular Engineering, Institute for Functional Intelligent Materials (I-FIM), National University of Singapore, Singapore, 119077 Singapore; 7 Physics Department and LBNL, University of California, Emeritus, Berkeley, CA 94720 USA; 8 Paris Centre for Cosmological Physics, CNRS, Université de Paris, Emeritus, Astroparticule Et Cosmologie, F-75013 Paris, France; 9 Department of Physics, The Hong Kong University of Science and Technology, Emeritus, Clear Water Bay, Kowloon, Hong Kong

**Keywords:** Renewable energy, Energy science and technology, Materials science

## Abstract

The term 'Solar Cell’ is commonly used for Photovoltaics that convert light into electrical energy. However, light can be harvested from various sources not limited to the Sun. This work considers the possibility of harvesting photons from different star types, including our closest neighbor star Proxima Centauri. The theoretical efficiency limits of single junction photovoltaic devices are calculated for different star types at a normalized light intensity corresponding to the AM0 spectrum intensity with AM0 = 1361 W/m^2^. An optimal bandgap of > 12 eV for the hottest O5V star type leads to 47% Shockley-Queisser photoconversion efficiency (SQ PCE), whereas a narrower optimal bandgap of 0.7 eV leads to 23% SQ PCE for the coldest red dwarf M0, M5.5Ve, and M8V type stars. Organic Photovoltaics (OPVs) are the most lightweight solar technology and have the potential to be employed in weight-restricted space applications, including foreseeable interstellar missions. With that in mind, the Sun’s G2V spectrum and Proxima Centauri’s M5.5Ve spectrum are considered in further detail in combination with two extreme bandgap OPV systems: one narrow bandgap system (PM2:COTIC-4F, *E*_g_ = 1.14 eV) and one wide bandgap system (PM6:o-IDTBR, *E*_g_ = 1.62 eV). Semi-empirically modeled JV-curves reveal that the absorption characteristics of the PM2:COTIC-4F blend match well with both the G2V and the M5.5Ve spectrum, yielding theoretical PCEs of 22.6% and 12.6%, respectively. In contrast, the PM6:o-IDTBR device shows a theoretical PCE of 18.2% under G2V illumination that drops sharply to 0.9% under M5.5Ve illumination.

## Introduction

After the discovery of the photoelectric effect in 1839 by Becquerel, the first patents for solar cells (U.S. 389,124 and US389125) were issued to Ed. Weston in 1888, laying the groundwork for off-grid electricity generation from the Sun’s abundant energy supply^1,2^. Intense research efforts at Bell Labs followed in the 1950s, which led to the first photovoltaic cell based on Si, reaching PCEs of 4% in 1953^3^. PCEs steeply increased in a few years to 14% (1960) and the first applications of PVs were explored in satellite and space techologies^4^.

PV research has evolved dramatically since then and has breached into a whole spectrum of different technologies, including inorganic single-crystal and polycrystalline Si cells, GaAs-based cells, polycrystalline thin-film photovoltaics, as well as perovskite and organic photovoltaics (OPVs)^5–7^. Single crystal Si cells have turned into an economically viable sustainable energy harvesting solution, featuring PCEs of over 25% and long-term stability of 25 + years at costs that have steadily declined in the past decade^8,9^. Besides utility-scale power generation, they serve off-grid applications such as parking meters, portable chargers, or street lights and can be integrated into buildings, greenhouses or vehicles, and serve electricity generation, water and waste water treatment in developing countries^10–18^. In addition to that, solar cells remain crucial for space applications and they are used on the International Space Station (ISS) or to power moon and mars rangers^19,20^.

While the realization of such applications has pushed the frontiers of space exploration within our solar system in the 1960s and 1970s, many open scientific questions remain, such as the possibility of extra-terrestrial life. The first extrasolar planets were discovered in 1992, followed by the breakthrough discovery of 51 Pegasi b orbiting the Sun-like star 51 Pegasi in 1995 (awarded with the Nobel Prize in Physics 2019)^21,22^. As of today, more than 4000 exoplanets have been discovered thanks to advanced space telescopes such as Kepler ^23,24^, TESS ^25^, and CHEOPS^26^. The discoveries suggest that there could be ~ 100 billion Earth-like planets throughout our galaxy alone that may offer habitable conditions^27^.

One exciting target for exploration is the planet *Proxima Centauri b*. It is part of the Alpha Centauri star system and is located at a distance of 4.2 light years from Earth. The planet orbits the red dwarf star *Proxima Centauri*, the closest star to our planet. Proxima Centauri b is in its habitable zone, at a distance of about 7.5 million km, and has an orbital period of 11.2 days^28^. The *Breakthrough Starshot* initiative aims to send thousand centimeter-sized, gram-scale probes mounted on a spacecraft with ‘light sails’ to the Alpha Centauri system to gather and signal back data to the Earth^29–31^. Such light sails accelerate via light propulsion, the concept of using the momentum of the light, sometimes referred to as radiation pressure^32–34^. The sails’ technological roadmap is still under development but it is well understood that the acceleration is limited by the ratio of flux to mass per unit area^35^. Thus, the sails are supposed to be lightweight in order to be accelerated by earth-based lasers to a speed of ~ 0.2c, which would allow the telescope to reach the Alpha Centauri within decades^30,36–38^. Such acceleration is only possible when combining powerful GW lasers with a very lightweight spacecraft of a few grams. Currently, the scientific community is exploring the technical feasibility of the project, and addressing thermal management and the light sail design^30,31,38,39^.

This work addresses the possibility of harvesting photon energy from different types of stars, including Proxima Centauri. While technical details need to be worked further, it is likely that the strict weight requirements render inorganic solar cells based on Si or GaAs unsuitable. Moreover, the light sails need to be significantly curved, with an increased curvature under acceleration^30^. This requirement also makes organic photovoltaics (OPVs) prime candidates as their photoactive layers are nanometer-thin films that can be deposited on thin plastic substrates and on curved surfaces^15,40,41^. Further, they exhibit the required mechanical flexibility^41–43^.

In the case of the *Breakthrough Starshot* initiative, the laser-facing side of the light sails has to be made from a reflective and heat-resistant material^38^. It may be possible to use the parabolic light sail as a starlight concentrator to provide sufficient irradiation of a photovoltaic device placed in its focus even far from the star. The light sail design is not very well defined at this point, so integrating extremely lightweight OPVs directly into its multilayered structure remains a possibility^44^. Moreover, conformable OPVs could be integrated into interstellar probes, satellites, and spaceships or be transported for applications on colonized exoplanets in the distant future. While technologically still challenging, the exploration of the interstellar space marks the next phase of space exploration and is considered to be within reach in the next century. Herein, we first address fundamental limitations of photovoltaics for interstellar applications. In the second part of the paper, we focus the reader’s attention on the possibilities for OPVs in interstellar applications. In particular, we demonstrate how the inherent material properties of organic semiconductor donor:acceptor bulk-heterojunction (BHJ) active layers influence their potential to be employed near Proxima Centauri.

## Result and discussion

### Stellar types and their spectra

**Figure 1:**
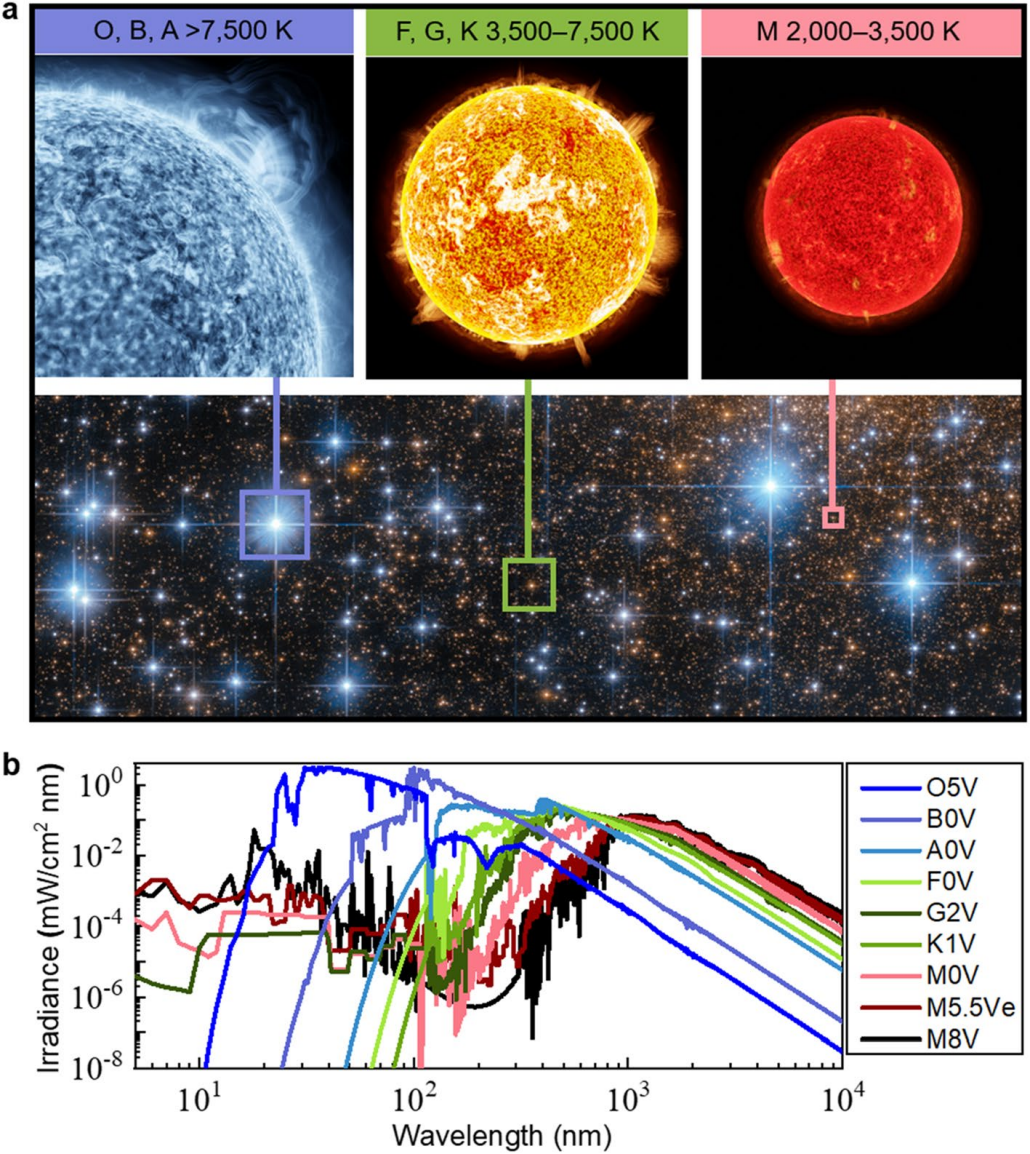
Stellar types and their spectra. **a** Visually representative examples of O, B, A, F, G, K and M stellar types (hot to cold) within our galaxy (source: NASA54). **b** Spectral distributions of stars ranging from O5V to M8V.

‘Solar cells’ is a commonly established term for PV devices that interconvert light and electrical energy, because, so far, they typically harvest Sun light. The research and development of terrestrial PVs typically considers the standard solar spectrum AM1.5, which has an integrated intensity of 1000 W/m^2^. Near Earth’s orbit-based PVs are located outside the atmosphere and are exposed to the standard AM0 solar irradiance spectrum with an incident intensity of 1361 W/m^2^^45^. The AM0 irradiance spectrum is defined by the Sun’s surface temperature and the distance between the Earth and the Sun. With a surface temperature close to 6,000 K, the Sun is classified as a G2V star, according to the main sequence of stars. In this work, we consider spectra from stars of types O5V, B0V, A0V, F0V, G2V, K0V, M5.5Ve, and M8V, representing examples from all major stellar spectral types^46,47^. The M5.5Ve and M8V stars are the Proxima Centauri and TRAPPIST-1 stars, the latter being an ultra-cool red dwarf star 40.7 light years away hosting at least seven planets, some of which are in the habitable zone, where water can exist in liquid form^48–50^. Figure 1a shows the different start types, together with their temperatures, and Fig. 1b provides different stellar spectra. The spectra of the stars are obtained from the Pickles (1998) catalog ^51^, which provides spectral energy distributions from 115 to 2500 nm for a wide range of stellar types. For the O5V and B0V stars, which have significant emissions below 115 nm, the Pickles (1998) spectra were fitted with the OSTAR2002 ^52^ and BSTAR2006 ^53^ models below 115 nm, respectively. At longer wavelengths, the blackbody spectrum is employed. While approximate, the blackbody part of the spectrum contributes less than 1% of the overall energy. All spectra are normalized to an incident integrated intensity of 1361 W/m^2^ for direct comparison.

### Photovoltaic efficiency limits

**Figure 2:**
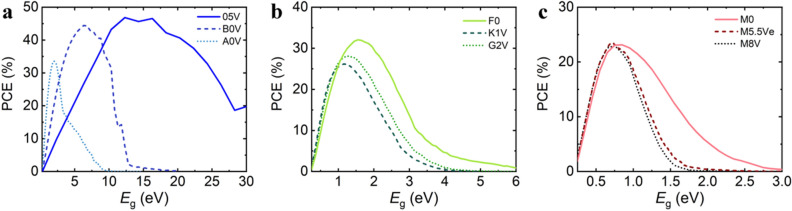
Theoretical photovoltaic performance limits for the different stellar types. **a** PCE as a function of the band gap for spectral star types O5V, B0V, and A0V. **b** PCE as a function of the band gap for spectral star types F0, K1V, and G2V. c) PCE as a function of the band gap for spectral star types M0, M5.5Ve, and M8V.

The theoretical performance limits of single-junction PVs under illumination with these different spectral star types were determined within the scope of the Shockley-Queisser limit model^54^, as shown in Fig. 2. In the first step, the photon flux *Φ* was calculated from the shown spectra as outlined in the Supporting Information. The theoretical photocurrent *J*_ph_ is obtained as a function of the band gap *E*_g_ based on the integration of the photon flux *Φ* multiplied with the elementary charge *q*, assuming the black body absorption:

1 $$ J_{{ph}}  = q\int\limits_{{E_{g} }}^{\infty } \Phi  (E)dE $$

The lower limit of recombination losses, also referred to as the radiative limit, is determined by bimolecular recombination processes that are inevitable for temperatures > 0 K^54,55^. The resulting minimum recombination current *J*_rec_ is obtained from detailed-balance calculations^54,55^ as:

2 $$  J_{{rec}}  = \frac{{2q\pi }}{{c^{2} h^{3} }}\int\limits_{{E_{g} }}^{\infty } {\frac{{E^{2} }}{{\exp \left( {\frac{{E - qV}}{{k_{B} T}}} \right) - 1}}} dE $$

Based on *J*_rec_, a theoretical *JV*-curve can be obtained for positive voltages (0 V to *E*_g_/*q*) as the sum of the photocurrent and the recombination current (note the negative sign of the photocurrent, which is reduced in its magnitude by adding the recombination current).

3 $$ J(V,E_{g} ) = J_{{ph}}  + J_{{rec}}  = q\left( {\int\limits_{{E_{g} }}^{\infty } {\Phi (E)dE + \frac{{2\pi }}{{c^{2} h^{3} }}} \int\limits_{{E_{g} }}^{\infty } {\frac{{E^{2} }}{{exp\left( {\frac{{E - qV}}{{k_{B} T}}} \right) - 1}}} dE} \right) $$

Having calculated the *JV-*curves as a function of *E*_g_, it is possible to determine the open circuit voltage *V*_oc_, the short-circuit current *J*_sc_ and the fill factor *FF* for each curve. The SQ PCE dependence on *E*_g_ values is then obtained as the product of these performance parameters per incident power as $PCE=\frac{{J}_{sc}\cdot {V}_{oc}\cdot FF }{{P}_{in}}$.

Figure 2a shows the resulting SQ PCEs that can be obtained for single-junction PV devices exposed to the spectra of hottest star types O5V, B0V and A0V, revealing maxima of 46.8% at 12.3 eV, 44.3% at 6.8 eV and 33.6% at 2 eV, respectively. These maxima can be considered as the optimum band gaps for any photovoltaic material to obtain the highest PCEs under the respective illumination conditions.

It should be mentioned that the optimal band gap of 12.3 eV for the hottest star type O5V is beyond a practical band gap range. For instance, the widest band gap polymorphs of classical dielectric materials *α*-Al_2_O_3_ and *β*-moganite SiO_2_ possess band gaps of 8.8 eV^56^ and 9.7 eV^57^, respectively. To our knowledge, there are no reports on using these dielectrics as the active material in any optoelectronic devices. However, the optimal band gap of 6.8 eV for the second hottest star type B0V already is in the range of extremely wide band gap optoelectronic materials. Diamond (*E*_g_ = 5.47 eV)^58^ and cubic Boron Nitride (*E*_g_ = 6.36 eV)^59^ have been successfully employed as photo-active materials in solar-blind deep-ultraviolet photodetectors^60–63^ and, thus, can potentially be used as efficient PV materials next to hot stars of the B0V type.

Figure 2b displays the SQ PCEs for the F0, G2V and K1V spectra, with maxima of 32.1% at 1.5 eV, 28.1% at 1.2 eV, and 26.2% at 1.15 eV respectively. Band gaps up to approximately 6 eV, 5 eV and 4 eV yield non-zero SQ PCEs. For the coldest spectral types M0, M5.5Ve and M8V, SQ PCE maxima of 23.1% at 0.85 eV, 23.4% at 0.72 eV, and 23.0% at 0.75 eV are determined with tails approaching zero around 3 eV, 2 eV and 1.5 eV, respectively, as shown in Fig. 2c.

To summarize these calculations, drastic changes of the SQ PCEs as well as the ideal band gap can be observed depending on the star type and its spectral distribution. Having elucidated how the SQ limits depend on the band gap and on the spectral type, we will now focus on a specific PV material class. As outlined above, the strict weight requirements allow only for thin-film PVs. Among these technologies are Cadmium-Telluride, Cupper-Indium-Gallium-Selenide (CIGS), Perovskites and Organic PVs^64–66^. The latter represents the most lightweight solution thanks to ultra-thin photoactive layers of about 100 nm that are comprised of low density carbon-based molecular materials^67–69^. Due to their molecular tunability, a wide range of band gaps is accessible with OPVs, which is of particular interest for the application under different illumination conditions, such as in the proximity of different stars. For these reasons, we explore the commercially available extremely narrow or wide band gap lightweight OPVs next to the Sun (G2V spectrum) and near Proxima Centauri (M5.5Ve spectrum).

### Performance of narrow and wide band gap OPV systems near the sun and proxima centauri

**Figure 3:**
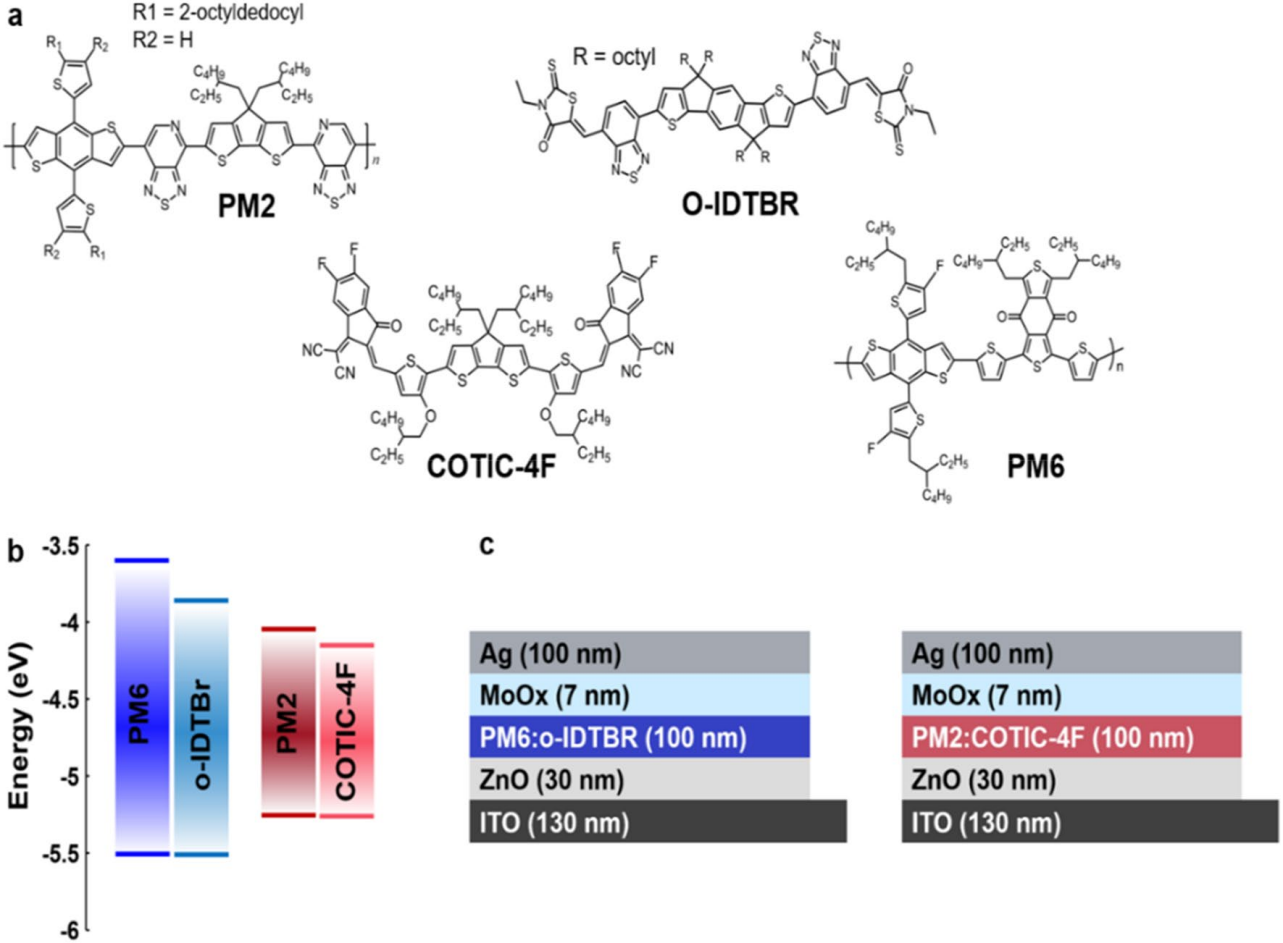
OPV model systems. **a** Chemical structures of the organic semiconducting donor polymers PM6 and PM2 as well as of the NFAs O-IDTBR and COTIC-4F. **b** Energy diagram for the materials. **c** Schematic device structure for inverted solar cells based on PM6:o-IDTBR and PM2:COTIC-4F active layers.

Figure 3a displays the chemical structures of the selected organic semiconductor donor polymers PM2 and PM6 and of the small-molecule non-fullerene acceptors (NFAs) o-IDTBR and COTIC-4F. Figure 3b shows their lowest unoccupied molecular orbitals (LUMOs) and highest occupied molecular orbitals (HOMOs), which may be considered as equivalents to the bottom of the conduction band and top of the valance band, respectively. The values were taken from the literature. ^70–73^ The wide-band gap components PM6:o-IDTBR and the narrow band gap components PM2:COTIC-4F are paired and employed using an inverted device structure (ITO/ZnO/active layer/MoO_x_/Ag), as shown schematically in Fig. 3c.

**Figure 4:**
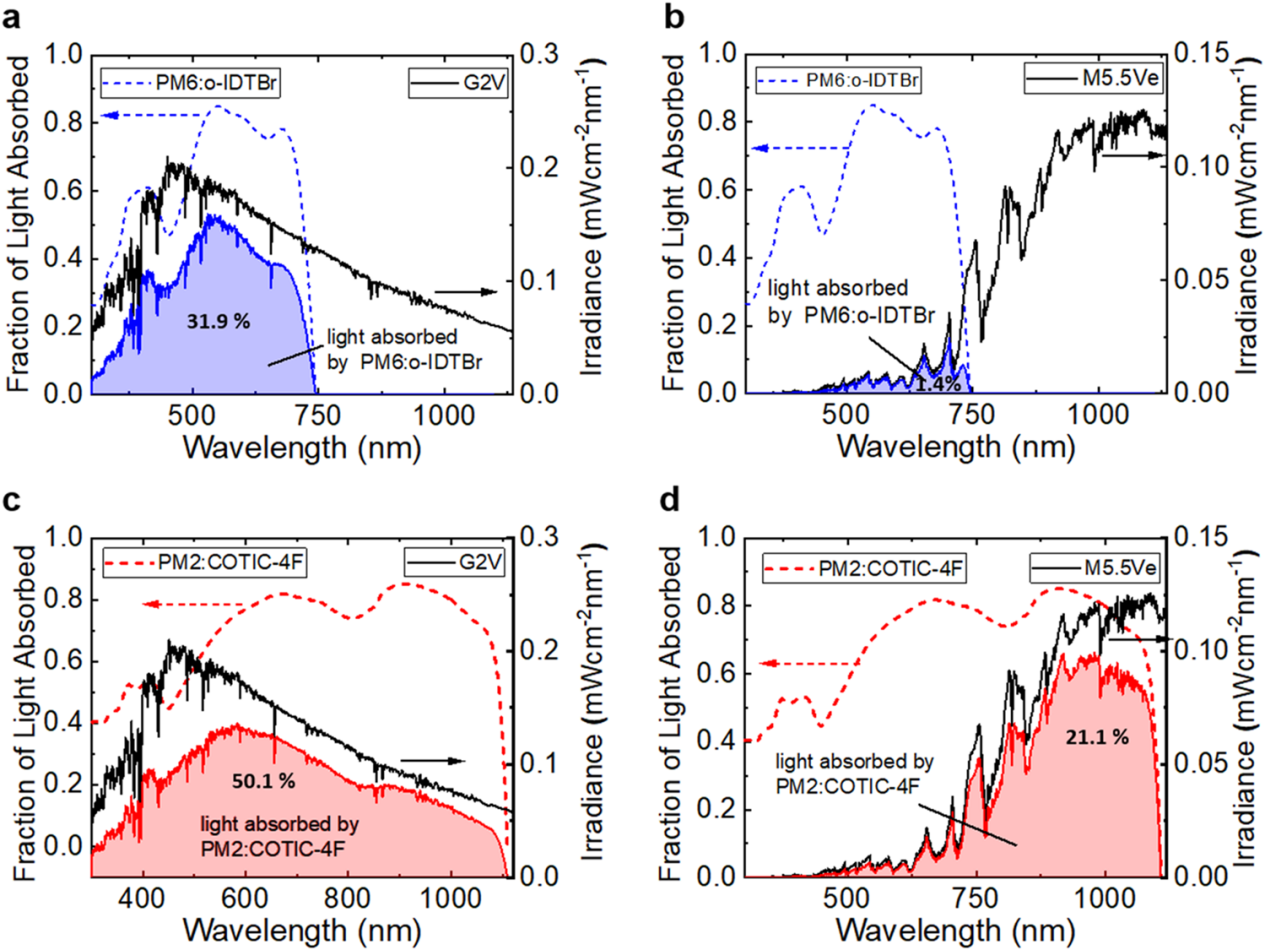
Comparison of the spectral match of the G2V and M5.5Ve spectra with two BHJ OPV blends. **a** G2V spectrum (black) and the fraction of light absorbed by PM6:o-IDTBr (blue dashed) and the amount of light absorbed (blue area). **b** M5.5Ve spectrum (black) and the fraction of light absorbed by PM6:o-IDTBr (blue dashed) and the amount of light absorbed (blue area). c) G2V spectrum (black) and the fraction of light absorbed by PM2:COTIC-4F (red dashed) and the amount of light absorbed (red area). d) M5.5Ve spectrum (black) and the fraction of light absorbed by PM2:COTIC-4F (red dashed) and the amount of light absorbed (red area).

The wide band gap PM6:o-IDTBR system features an effective band gap (LUMO_A_—HOMO_D_) of 1.62 eV, contrasting the narrow effective band gap of only 1.14 eV of the PM2:COTIC-4F blend. Based on this difference, altered absorption characteristics are expected with favored IR-absorption in the narrow band gap system. Experimentally measured values for the refractive index *n*(λ) and the extinction coefficient *k*(λ) of the bulk-heterojunction active layers are given in the Supporting Information. Figure 4 provides insight into how the absorption characteristics of the two blends match the spectral irradiance of the M5.5Ve Proxima Centauri and the G2V Sun spectrum.

Figure 4a and b reveal the spectral match of PM6:o-IDTBR (blue) solar cells with G2V and M5.5Ve illumination, respectively. The spectral irradiance of the Sun and of Proxima Centauri are shown in black (right axis), respectively. The dashed lines represent the fraction of the light that is absorbed by the photo-active layer under the different illumination in the above-mentioned inverted device configurations (Fig. 1), which was obtained from optical Transfer Matrix Simulations, taking into account all device layers and their thicknesses.^74,75^ The net amount of light absorbed corresponds to the integrated area under the curve (blue/red areas), which is the product of their spectral irradiance and the fraction of light absorbed.

It becomes apparent that the wide band gap system absorbs a significantly larger amount of light under G2V illumination than under M5.5Ve illumination. Under G2V illumination, 31.9% of the light is absorbed thanks to a good overlap of the G2V spectrum and the absorption characteristics of PM6:o-IDTBR below 700 nm. In contrast, under the red-shifted M5.5Ve illumination these absorption features lead to a large spectral mismatch and only 1.4% of the light can be absorbed.

Figure 4c and d show the same information for the PM2:COTIC-4F system. Thanks to the strong irradiance tail of the G2V spectrum that stretches beyond 1000 nm, the PM2:COTIC-4F system demonstrates good overlap as well, and 50.1% of the light is absorbed by the narrow band gap OPV. In contrast to the wide band gap system, a good spectral match of the absorption characteristics is found under the M5.5Ve spectrum as well, with a peak irradiance around 1100 nm, leading to 21.1% of the photons being absorbed.

Lastly, we estimate the effect of the spectral overlap of the two OPV blends under M5.5Ve and G2V illumination on device performance. The *JV*-curves are calculated within the scope of a semiempirical model as the sum of the theoretical short circuit current *J*_sc,theo_ and the voltage-dependent recombination current *J*_rec._ The *J*_sc,theo_ was obtained from optical Transfer Matrix Simulations under M5.5Ve and G2V illumination, following Eq. 5. The spatially and wavelength-dependent generation rate *G*(x, λ) of photo-generated charge carriers in the active layer was obtained based on the device structures shown in Fig. 3c, taking into account the complex interference pattern that is determined by the thicknesses and the optical constants of all layers, as outlined in our previous work. ^75–77^.

*J*_rec_ was calculated according to Eq. 6 through 9, with *q* being the elementary charge, *k*_bm_ the bimolecular recombination coefficient, *n* the charge carrier density, *T* the temperature assumed as 298 K, *N*_c_ the effective density of states, *E*_g_ the effective band gap (LUMO_A_—HOMO_D_) and *V* the voltage. The used values of the considered parameters are chosen in such, that they are in the physically-based range for OPV materials, and are given in Table 1.^78–80^.

4 $$ J = J_{sc,theo} + J_{rec} $$

5 $$ J_{sc,theo} = q \cdot P_{g} \int\limits_{{}}^{\lambda } {\int\limits_{{}}^{L} {G(x,\lambda )dxd} } \lambda $$

6 $$ J_{{rec}}  = qLk_{{bm}} n(V)^{2}  $$

With the definition of *n* and *n*_i_.

7 $$ n(V)^{2} = n_{i}^{2} exp\left( {\frac{qV}{{k_{B} T}}} \right) $$

8 $$ n_{i} = N_{c}^{2} exp\left( {\frac{{ - E_{g} }}{{k_{B} T}}} \right) $$

We obtain.

9 $$  J_{{rec}}  = qLk_{{bm}}  \cdot exp\left( { - \frac{{E_{g} }}{{k_{B} T}}} \right)exp\left( {\frac{{qV}}{{k_{B} T}}} \right)  $$

**Table 1:** Parameters used for the calculation of the JV curves for PM2:COTIC-4F and PM6:o-IDTBR.

Parameter	Value PM2:COTIC-4F, PM6:o-IDTBR
Effective band gap* E*_g_	1.14 eV, 1.62 eV
Effective density of States* N*_c_	2.5 × 10^19^ cm^-3^
Geminate recombination prefactor *P*_g_	1
Bimolecular recombination coefficient *k*_bm_	5 × 10^–11^ cm^3^/s
Temperature* T*	298 K

**Figure 5:**
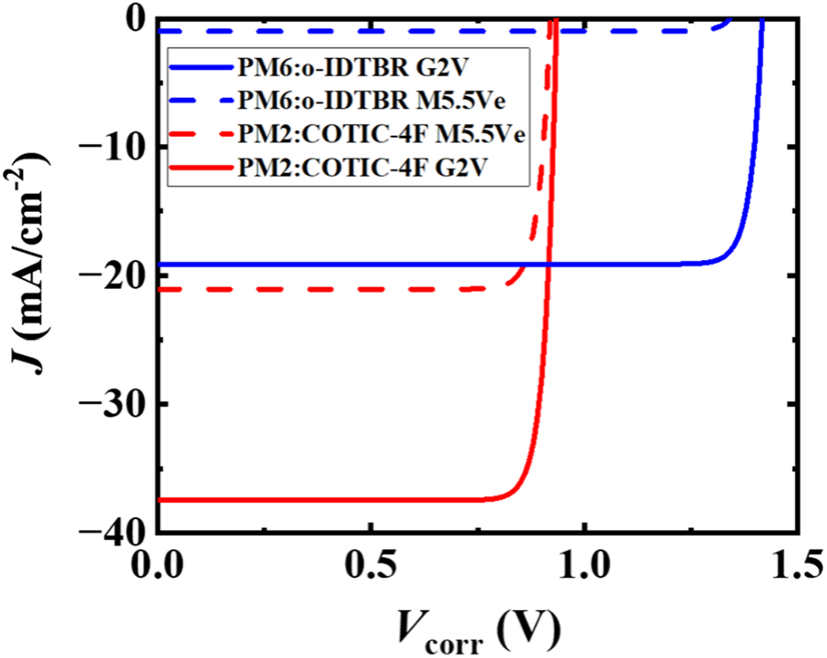
*JV*-characteristics of OPVs with PM6:o-IDTBR (blue) and PM2:COTIC-4F (red) active layers under M5.5Ve (dashed line) and G2V (solid lines) illumination.

The resulting *JV* curves are shown in Fig. 5. We note that this approach disregards possible energy losses and complex recombination processes, including geminate recombination losses (*P*_g_ assumed to be unity), as well as losses via surface and bulk trap-assisted recombination mechanisms.^81–83^ Nevertheless, this approach serves as a first approximation to compare the OPV’s potential in the environment close to the Sun or Proxima Centauri.

First, we observe that both systems perform lower under M5.5Ve illumination. The reduced performance originates from a reduced short-circuit current in the narrow band gap PM2:COTIC-4F system. In the wide bandgap system, it originates from both a reduced short-circuit current and a reduced *V*_oc_.

The wide-band gap system PM6:o-IDTBR yields a simulated *J*_sc_ of -19.1 mA/cm^2^, a *V*_oc_ of 1.42 V, a *FF* of 0.91 and a PCE of 18.2% under G2V illumination. In contrast, in the environment close to Proxima Centauri, a *J*_sc_ of only 1.0 mA/cm^2^ would be obtained and a noticeable reduction in the *V*_oc_ to 1.34 V can be observed. With a *FF* of 0.91 a low theoretical PCE of only 0.9% is obtained, representing a PCE drop to only about 5% of the PCE under G2V illumination. We can summarize that the wide-band gap system shows more pronounced changes depending on the illumination due to the large difference in the spectral overlap. The PM2:COTIC-4F OPV yields a *J*_sc_ of 37.6 mA/cm^2^, a *V*_oc_ of 0.93 V, a *FF* of 0.88 and a theoretical PCE of 22.6% under G2V illumination. Under M5.5Ve illumination, the *V*_oc_ is almost as high as under the Sun spectrum with a value of 0.92 V, however, the *J*_sc_ is reduced to 21.3 mA/cm^2^. With an *FF* of 0.88, the narrow band gap OPVs still exhibit a theoretical PCE of 12.6%, maintaining 56% of the PCE under G2V illumination. Therefore, we can conclude that the narrow band gap system is a more suitable choice for any future application in proximity to Proxima Centauri. The application of OPVs for interstellar use once more demonstrates the benefits that arise from the flexible band gap tuning due to the molecular nature of organic semiconductors and the chemical diversity it affords. We note that while the band gap engineering of OPVs underwent great success, smaller band gaps below 1.1 eV have not been accessible yet. Therefore, a narrow band gap system based on COTIC-4F or its derivatives offers one of the most promising spectral matches for use near Proxima Centauri to date. The details a summarized in Table 2.

**Table 2:** Performance parameters for the two OPV structures under illumination conditions representing the environment next to the sun (G2V) and Proxima Centauri (M5.5Ve).

System	*J*_sc_ (mA/cm^2^)	*V*_oc_ (V)	FF	PCE (%)
PM6:o-IDTBR G2V	−19.1	1.42	0.91	18.2
PM6:o-IDTBR M5.5Ve	−1.0	1.34	0.91	0.9
PM2:COTIC-4F G2V	−37.6	0.93	0.88	22.6
PM2:COTIC-4F M5.5Ve	−21.3	0.92	0.88	12.6

## Conclusion

The spectral distribution of stars spans a wide range depending on their temperature. This work has laid out how the spectral distributions impact the band gap-dependent SQ limits and has determined the SQ performance limits for the hottest O5V type stars to be about 47% at band gaps of 12–16 eV. Colder stars require narrower band gaps as low as 0.7 eV to reach SQ PCEs of 23%.

In the second part of this work, the application of a wide band gap and a narrow band gap OPV system was considered, in the first case near the Sun (G2V type star) and in the other case near the red dwarf star Proxima Centauri (M5.5Ve type star) that is the star closest to Earth. The wider band gap system shows a theoretical PCE of 18.2% in G2V illumination that drops sharply to 0.9% under M5.5Ve illumination due to a poor spectral overlap of the absorption and the M5.5Ve spectrum. In contrast, the more extensive spectral overlap found for the PM2:COTIC-4F narrow band gap system for both the G2V and the M5.5Ve spectra leads to theoretical PCEs of 22.6% and 12.6%, respectively. Our results demonstrate the need for narrow band gap systems for interstellar OPV applications near Proxima Centauri, or, more generally, the need to consider the spectral irradiance and the material properties of OPVs for interstellar applications.

We note that OPVs are not as robust and established yet as their inorganic counterparts. However, research in the OPV field is currently addressing the remaining long-term stability challenges via the molecular design of new donors and acceptors, the incorporation of cross-linker and the development of thermodynamically stable morphological blends. These efforts are expected to result in significant improvements of OPV long-term stability for future space applications.

## Material and method

### Organic bulk-heterojunction active layers processing

The glass substrates were cleaned with detergent, then subsequently ultra-sonicated in acetone and isopropanol, and dried at 100 °C. Afterward, the glass substrates undergo a UV-ozone treatment for 15 min.

PM2:COTIC-4F (mass ratio 1:1.5 with total concentration of 20 mg/ml) was dissolved in chlorobenzene with 1 vol% 1-chloronaphthalene. The solution was stirred at 60 °C for at least 8 h. Active layers were spin-coated at 1200 rpm on the glass substrates in a dry nitrogen atmosphere. PM6:o-IDTBR (mass ratio 1:1 with total concentration of 20 mg/ml) was dissolved in chlorobenzene with 1 vol% 1-chloronaphthalene. The solution was stirred at 60 °C for at least 8 h. Active layers were spin-coated at 2000 rpm the glass substrates in a nitrogen-filled glovebox.

### Optical properties and optical simulations

The optical properties of the photoactive blends were obtained experimentally from transmission and reflection measurements. The transmission and reflection spectra of the D:A bulk-heterojunction films were measured using a UV–vis-NIR spectrophotometer (Lambda 1050, Perkin Elmer). The thicknesses of the films were measured using a profilometer (Dektak XT Stylus Profiler). Modeling beyond the TMM method was carried out in python code based on the equations presented in the manuscript.

Optical Simulations were carried out using the freely available Transfer Matrix Method software and the optical properties of the electrode materials were used as reported in previous work.^74,75^.

### Supplementary Information

Supplementary Information.

## Data Availability

The materials and data that support the findings of this study are available from the corresponding authors on request.

## References

[1] Weston, E. UNITED STATES PATENT OFFICE. 3 (1888).

[2] Zaidi, B. *Solar Panels and Photovoltaic Materials*. (BoD – Books on Demand, 2018).

[3] Tress, W. *Organic Solar Cells: Theory, Experiment, and Device Simulation*. (Springer, 2014).

[4] *High-efficient low-cost photovoltaics: recent developments*. (Springer, 2009).

[5] NREL. Best Research-Cell Efficiency Chart. https://www.nrel.gov/pv/cell-efficiency.html (2022).

[6] Marques Lameirinhas RA, Torres JPN, de Melo Cunha JP (2022). A Photovoltaic technology review: history. Fundament. Appl. Energ..

[7] Dambhare MV, Butey B, Moharil SV (2021). Solar photovoltaic technology: a review of different types of solar cells and its future trends. J. Phys.: Conf. Ser..

[8] Documenting a Decade of Cost Declines for PV Systems. https://www.nrel.gov/news/program/2021/documenting-a-decade-of-cost-declines-for-pv-systems.html (2021).

[9] Ballif C, Haug F-J, Boccard M, Verlinden PJ, Hahn G (2022). Status and perspectives of crystalline silicon photovoltaics in research and industry. Nat. Rev. Mater..

[10] Alami AH (2022). Novel and practical photovoltaic applications. Therm. Sci. Eng. Prog..

[11] Brus VV (2019). Solution-processed semitransparent organic photovoltaics: from molecular design to device performance. Adv. Mater..

[12] Egbon, C., Oyekola, A. & Lie, T.-T. Design of stand alone photovoltaic system in developing countries: a case study of Kano, Nigeria. İn: 2018 Australasian Universities Power Engineering Conference (AUPEC) 1–6 (2018). doi:10.1109/AUPEC.2018.8757895.

[13] Esmailzadeh M, Noori S, Aliahmadi A, Nouralizadeh H, Bogers M (2020). A functional analysis of technological innovation systems in developing countries: an evaluation of Iran’s photovoltaic innovation system. Sustainability.

[14] Mamun MAA, Dargusch P, Wadley D, Zulkarnain NA, Aziz AA (2022). A review of research on agrivoltaic systems. Renew. Sustain. Energy Rev..

[15] Waller R, Kacira M, Magadley E, Teitel M, Yehia I (2022). Evaluating the performance of flexible, semi-transparent large-area organic photovoltaic arrays deployed on a greenhouse. AgriEngineering.

[16] Xue J (2017). Photovoltaic agriculture - New opportunity for photovoltaic applications in China. Renew. Sustain. Energy Rev..

[17] Ravishankar E (2021). Balancing crop production and energy harvesting in organic solar-powered greenhouses. Cell Rep. Phys. Sci..

[18] Ravishankar E (2020). Achieving net zero energy greenhouses by integrating semitransparent organic solar cells. Joule.

[19] NREL. Photovoltaic Applications. https://www.nrel.gov/pv/applications.html (2022).

[20] Garcia, M. About the Space Station Solar Arrays. *NASA* http://www.nasa.gov/mission_pages/station/structure/elements/solar_arrays-about.html (2017).

[21] Wolszczan A, Frail DA (1992). A planetary system around the millisecond pulsar PSR1257 + 12. Nature.

[22] Mayor M, Queloz D (1995). A Jupiter-mass companion to a solar-type star. Nature.

[23] Borucki WJ (2010). Kepler planet-detection mission: introduction and first results. Science.

[24] Howell SB (2014). The K2 mission: characterization and early results. Publ. Astron. Soc. Pac..

[25] Ricker, G. R. *et al.* The Transiting Exoplanet Survey Satellite. in (eds. MacEwen, H. A. et al.) 99042B (2016). doi:10.1117/12.2232071.

[26] Cessa, V. CHEOPS Mission overview. İn: International Conference on Space Optics — ICSO 2018 (eds. Karafolas, N., Sodnik, Z. & Cugny, B.) 128 (SPIE, 2019). doi:10.1117/12.2536048.

[27] Howell SB (2020). The grand challenges of exoplanets. Front. Astron. Space Sci..

[28] Anglada-Escudé G (2016). A terrestrial planet candidate in a temperate orbit around Proxima Centauri. Nature.

[29] Falcigno, O. Breakthrough Starshot: reaching for the stars. https://lweb.cfa.harvard.edu/~loeb/Loeb_Starshot.pdf (2022).

[30] Campbell MF, Brewer J, Jariwala D, Raman AP, Bargatin I (2022). Relativistic light sails need to billow. Nano Lett..

[31] Brewer J (2022). Multiscale photonic emissivity engineering for relativistic Lightsail thermal regulation. Nano Lett..

[32] Levchenko I, Bazaka K, Mazouffre S, Xu S (2018). Prospects and physical mechanisms for photonic space propulsion. Nature Photon.

[33] Poupko VY, Dyachenko PP, Gulevich AV, Ovcharenko MK, Zrodnikov AV (1999). Light propulsion for space flight. AIP Conference Proceed..

[34] Zhang T (2015). Macroscopic and direct light propulsion of bulk graphene material. Nature Photon..

[35] Hall, L. A Breakthrough Propulsion Architecture. *NASA* http://www.nasa.gov/directorates/spacetech/niac/2018_Phase_I_Phase_II/Breakthrough_Propulsion_Architecture_for_Interstellar_Precursor_Missions (2018).

[36] Kipping D (2017). Relativistic Light Sails. AJ.

[37] Heller R, Hippke M (2017). Deceleration of high-velocity interstellar photon sails into bound orbits at *α* Centauri. ApJ.

[38] Lubin P, Cohen AN, Erlikhman J (2022). Radiation effects from the interstellar medium and cosmic ray particle impacts on relativistic spacecraft. ApJ.

[39] Lien MR (2022). Experimental characterization of a silicon nitride photonic crystal light sail. Opt. Mater. Exp..

[40] Kaltenbrunner M (2012). Ultrathin and lightweight organic solar cells with high flexibility. Nat. Commun..

[41] Kaltenbrunner M (2012). Ultrathin and lightweight organic solar cells with high flexibility. Nat. Commun..

[42] Choi S (2015). ITO-free large-area flexible organic solar cells with an embedded metal grid. Org. Electron..

[43] Li Y, Xu G, Cui C, Li Y (2018). Flexible and semitransparent organic solar cells. Adv. Energy Mater..

[44] Santi G (2022). Multilayers for directed energy accelerated lightsails. Commun. Mater..

[45] Landis, G. *Space Photovoltaic Research and Technology 1995*. (NASA, Lewis Research Center, 1996).

[46] Gray, R. O., Corbally, C. J. & Burgasser, A. J. *Stellar Spectral Classification*. (Princeton University Press, 2009).

[47] Kaler, J. B. *Stars and Their Spectra: An Introduction to the Spectral Sequence*. (Cambridge University Press, 1997).

[48] Gillon M (2016). Temperate earth-sized planets transiting a nearby ultracool dwarf star. Nature.

[49] Gillon M (2017). Seven temperate terrestrial planets around the nearby ultracool dwarf star TRAPPIST-1. Nature.

[50] Delrez L (2018). Early 2017 observations of TRAPPIST-1 with Spitzer. Mon. Not. R. Astron. Soc..

[51] Pickles AJ (1998). A stellar spectral flux library: 1150–25000 Å. PUBL ASTRON SOC PAC.

[52] Lanz T, Hubeny I (2003). A grid of non-LTE line-blanketed model atmospheres of O-type stars. ASTROPHYS J SUPPL S.

[53] Lanz T, Hubeny I (2007). A grid of NLTE line-blanketed model atmospheres of early B-type stars. ASTROPHYS J SUPPL S.

[54] Shockley W, Queisser HJ (1961). Detailed balance limit of efficiency of p-n junction solar cells. J. Appl. Phys..

[55] Röhr JA, Lipton J, Kong J, Maclean SA, Taylor AD (2020). Efficiency limits of underwater solar cells. Joule.

[56] Filatova EO, Konashuk AS (2015). Interpretation of the changing the band gap of Al2O3 depending on its crystalline form: connection with different local symmetries. J. Phys. Chem. C.

[57] Güler E, Uğur G, Uğur Ş, Güler M (2020). A theoretical study for the band gap energies of the most common silica polymorphs. Chin. J. Phys..

[58] Wort CJH, Balmer RS (2008). Diamond as an electronic material. Mater. Today.

[59] Evans DA (2008). Determination of the optical band-gap energy of cubic and hexagonal boron nitride using luminescence excitation spectroscopy. J. Phys.: Condens. Matter.

[60] Lin C (2019). Diamond based photodetectors for solar-blind communication. Opt. Express, OE.

[61] Liao M (2021). Progress in semiconductor diamond photodetectors and MEMS sensors. Functional Diamond.

[62] Soltani A (2008). 193nm deep-ultraviolet solar-blind cubic boron nitride based photodetectors. Appl. Phys. Lett..

[63] Liu H (2018). High-performance deep ultraviolet photodetectors based on few-layer hexagonal boron nitride. Nanoscale.

[64] Liu Y (2023). Efficiency enhancement of copper indium gallium selenide solar cells fabricated on polyimide foils with multiple metal layers. Thin Solid Films.

[65] Ho-Baillie AWY (2022). Deployment opportunities for space photovoltaics and the prospects for perovskite solar cells. Adv. Mater. Technol..

[66] Doroody C, Rahman KS, Kiong TS, Amin N (2022). Optoelectrical impact of alternative window layer composition in CdTe thin film solar cells performance. Sol. Energy.

[67] Gao J (2022). Over 17.7% efficiency ternary-blend organic solar cells with low energy-loss and good thickness-tolerance. Chem. Eng. J..

[68] Zhao H (2022). Kinetics manipulation enables high-performance thick ternary organic solar cells via R2R-compatible slot-die coating. Adv. Mater..

[69] Schopp N (2022). Unraveling device physics of dilute-donor narrow-bandgap organic solar cells with highly transparent active layers. Adv. Mater..

[70] Bertrandie J (2022). The energy level conundrum of organic semiconductors in solar cells. Adv. Mater..

[71] Zhang S, Qin Y, Zhu J, Hou J (2018). Over 14% efficiency in polymer solar cells enabled by a chlorinated polymer donor. Adv. Mater..

[72] Liang R-Z (2018). Carrier transport and recombination in efficient “All-Small-Molecule” solar cells with the nonfullerene acceptor IDTBR. Adv. Energy Mater..

[73] Lee J (2018). Bandgap narrowing in non-fullerene acceptors: single atom substitution leads to high optoelectronic response beyond 1000 nm. Adv. Energy Mater..

[74] Burkhard GF, Hoke ET, McGehee MD (2010). Accounting for interference, scattering, and electrode absorption to make accurate internal quantum efficiency measurements in organic and other thin solar cells. Adv. Mater..

[75] Schopp N, Brus VV, Lee J, Bazan GC, Nguyen T-Q (2021). A Simple approach for unraveling optoelectronic processes in organic solar cells under short-circuit conditions. Adv. Energy Mater..

[76] Schopp N, Nguyen T-Q, Brus VV (2021). Optical expediency of back electrode materials for organic near-infrared photodiodes. ACS Appl. Mater. Interfaces.

[77] Schopp N, Brus VV, Nguyen T-Q (2021). On optoelectronic processes in organic solar cells: from opaque to transparent. Adv. Opt. Mater..

[78] Koster LJA, Mihailetchi VD, Ramaker R, Blom PWM (2005). Light intensity dependence of open-circuit voltage of polymer:fullerene solar cells. Appl. Phys. Lett..

[79] Vollbrecht J (2020). Design of narrow bandgap non-fullerene acceptors for photovoltaic applications and investigation of non-geminate recombination dynamics. J. Mater. Chem. C.

[80] Brus VV (2019). Hall of fame article: solution-processed semitransparent organic photovoltaics: from molecular design to device performance. Adv. Mater..

[81] Brus VV, Proctor CM, Ran NA, Nguyen T-Q (2016). Capacitance spectroscopy for quantifying recombination losses in nonfullerene small-molecule bulk heterojunction solar cells. Adv. Energy Mater..

[82] Brus VV (2021). Temperature and light modulated open-circuit voltage in nonfullerene organic solar cells with different effective bandgaps. Adv. Energy Mater..

[83] Schopp N (2021). Effect of palladium-tetrakis(Triphenylphosphine) catalyst traces on charge recombination and extraction in non-fullerene-based organic solar cells. Adv. Func. Mater..

