# Supplementary material for: Interstellar photovoltaics
Source: Sci Rep. 2023 Sep 26;13:16114. doi: 10.1038/s41598-023-43224-5 (PMC10522670; doi:10.1038/s41598-023-43224-5)
Supplement: Supplementary file 1 — Supplementary Information. [file 41598_2023_43224_MOESM1_ESM.docx]

**Supplementary Information**

**Interstellar Photovoltaics**

Nora Schopp^1^, Ernazar Abdikamalov^2,3^, Andrii I. Mostovyi^2,4^, Hryhorii P. Parkhomenko^2^, Mykhailo M. Solovan^5^, Ernest A. Asare^2^, Guillermo C. Bazan^6^*, Thuc-Quyen Nguyen^1^*, George F. Smoot^3,7,8,9^* and Viktor V. Brus^2^*

*^1^Center for Polymers and Organic Solids, Department of Chemistry and Biochemistry, University of California Santa Barbara (UCSB), Santa Barbara, CA 93106, USA*

*^2^Department of Physics, School of Sciences and Humanities, Nazarbayev University, 010000 Astana, Republic of Kazakhstan*

*^3^Energetic Cosmos Laboratory, Nazarbayev University, Astana 010000, Republic of Kazakhstan*

*^4^Department of Electronics and Energy Engineering, Yuriy Fedkovych Chernivtsi National University, Chernivtsi 58012, Ukraine*

*^5^* *Faculty of Physics, Adam Mickiewicz University, Poznan 61-614, Poland*

*^6^* *Departments of Chemistry and Chemical & Biomolecular Engineering, Institute for Functional Intelligent Materials (I-FIM), National University of Singapore, Singapore, 119077 Singapore*

*^7^Physics Department and LBNL, University of California, Berkeley, CA 94720 USA, emeritus*

*^8^Paris Centre for Cosmological Physics, Université de Paris, CNRS, Astroparticule et Cosmologie, Paris F-75013, France, emeritus*

*^9^Department of Physics, The Hong Kong University of Science and Technology, Clear Water Bay, Kowloon, Hong Kong, emeritus*

*^*^Correspondence should be addressed to Viktor V. Brus* (*email:* [*vvbrus@gmail.com*](mailto:vvbrus@gmail.com)), George F. Smoot (*email:* [*gfsmoot@lbl.gov*](mailto:gfsmoot@lbl.gov)), Thuc-Quyen Nguyen (*email:* [*quyen@chem.ucsb.edu*](mailto:quyen@chem.ucsb.edu)), Guillermo C. Bazan (*email:* [*chmbgc@nus.edu.sg*](mailto:chmbgc@nus.edu.sg))

**Schockley-Queisser Limit Calculation**

The photon flux φ(λ ) is obtained from the spectra shown in Figure 1 in the main text as:

$$\phi\left( \lambda\right)= I\left( \lambda\right)*\frac{\lambda}{h\cdot c}$$

The light intensity and the photon flux are both then converted to a function of the energy E in eV:

$$\phi\left( E \right)= \phi\left( \lambda\right)\cdot\frac{\lambda}{E}$$

$$I\left( E \right)=I\left( \lambda\right)\cdot\frac{\lambda}{E}$$

**Optical Properties**

The optical properties of ITO, ZnO, MoOx and Ag can be found in previous work.^[1]^ The optical properties of the active layers are shown in Figure S1.

**
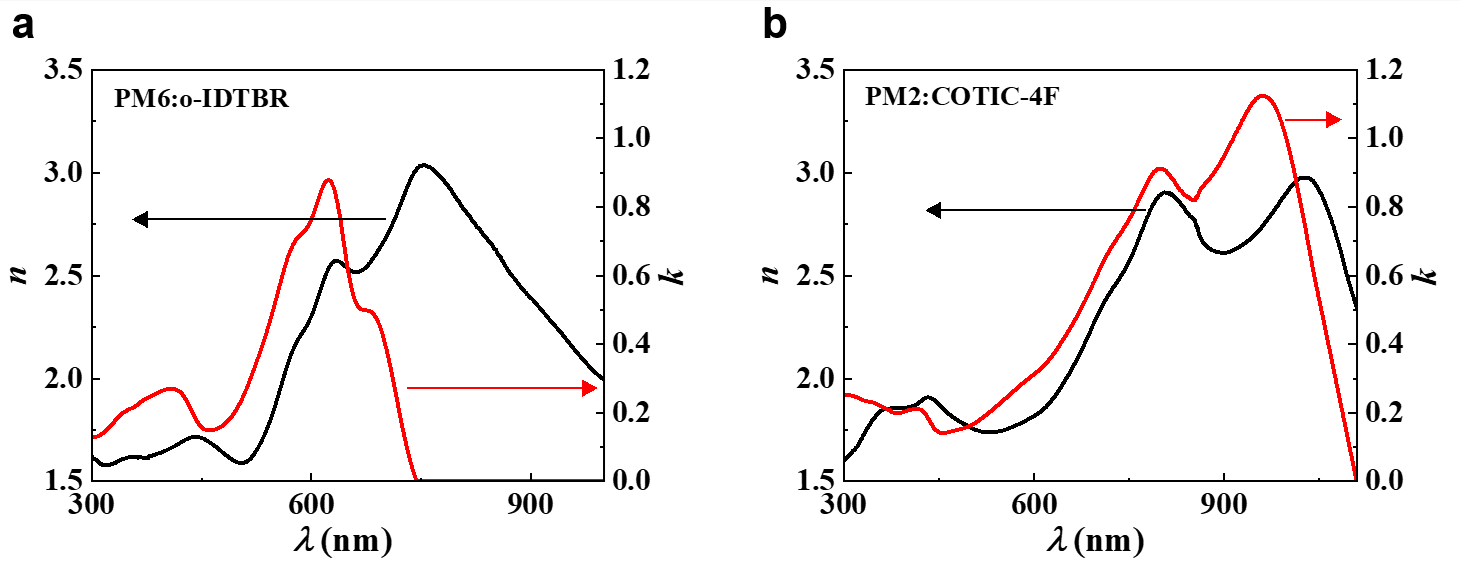
**

**Figure S1.** The refractive index *n* and extinction coefficient *k* for the two photoactive blends.

**Supporting References**

[1] N. Schopp, V. V. Brus, J. Lee, G. C. Bazan, T.-Q. Nguyen, *Advanced Energy Materials* **2021**, *11*, 2002760.
